# Whisking, Sniffing, and the Hippocampal θ-Rhythm: A Tale of Two Oscillators

**DOI:** 10.1371/journal.pbio.1002385

**Published:** 2016-02-18

**Authors:** David Kleinfeld, Martin Deschênes, Nachum Ulanovsky

**Affiliations:** 1 Department of Physics, University of California, San Diego, La Jolla, California, United States of America; 2 Section of Neurobiology, University of California, San Diego, La Jolla, California, United States of America; 3 Department of Psychiatry and Neuroscience, Laval University, Québec City, Canada; 4 Department of Neurobiology, Weizmann Institute of Science, Rehovot, Israel

## Abstract

The hippocampus has unique access to neuronal activity across all of the neocortex. Yet an unanswered question is how the transfer of information between these structures is gated. One hypothesis involves temporal-locking of activity in the neocortex with that in the hippocampus. New data from the Matthew E. Diamond laboratory shows that the rhythmic neuronal activity that accompanies vibrissa-based sensation, in rats, transiently locks to ongoing hippocampal θ-rhythmic activity during the sensory-gathering epoch of a discrimination task. This result complements past studies on the locking of sniffing and the θ-rhythm as well as the relation of sniffing and whisking. An overarching possibility is that the preBötzinger inspiration oscillator, which paces whisking, can selectively lock with the θ-rhythm to traffic sensorimotor information between the rat’s neocortex and hippocampus.

## Abstract 2

Transient phase locking of rhythmic sensory input with the hippocampal theta-rhythm may be a critical aspect of decision-making in a learned task. This Primer assesses the evidence for such dynamic gating. Read the associated Research Article.

The hippocampus lies along the margins of the cortical mantle and has unique access to neuronal activity across all of the neocortex. From a functional perspective, the hippocampus forms the apex of neuronal processing in mammals and is a key element in the short-term working memory, where neuronal signals persist for tens of seconds, that is independent of the frontal cortex (reviewed in [1,2]). Sensory information from multiple modalities is highly transformed as it passes from primary and higher-order sensory areas to the hippocampus. Several anatomically defined regions that lie within the temporal lobe take part in this transformation, all of which involve circuits with extensive recurrent feedback connections (reviewed in [3]) (Fig 1). This circuit motif is reminiscent of the pattern of connectivity within models of associative neuronal networks, whose dynamics lead to the clustering of neuronal inputs to form a reduced set of abstract representations [4] (reviewed in [5]). The first way station in the temporal lobe contains the postrhinal and perirhinal cortices, followed by the medial and lateral entorhinal cortices. Of note, olfactory input—which, unlike other senses, has no spatial component to its representation—has direct input to the lateral entorhinal cortex [6]. The third structure is the hippocampus, which contains multiple substructures (Fig 1).

**Fig 1:**
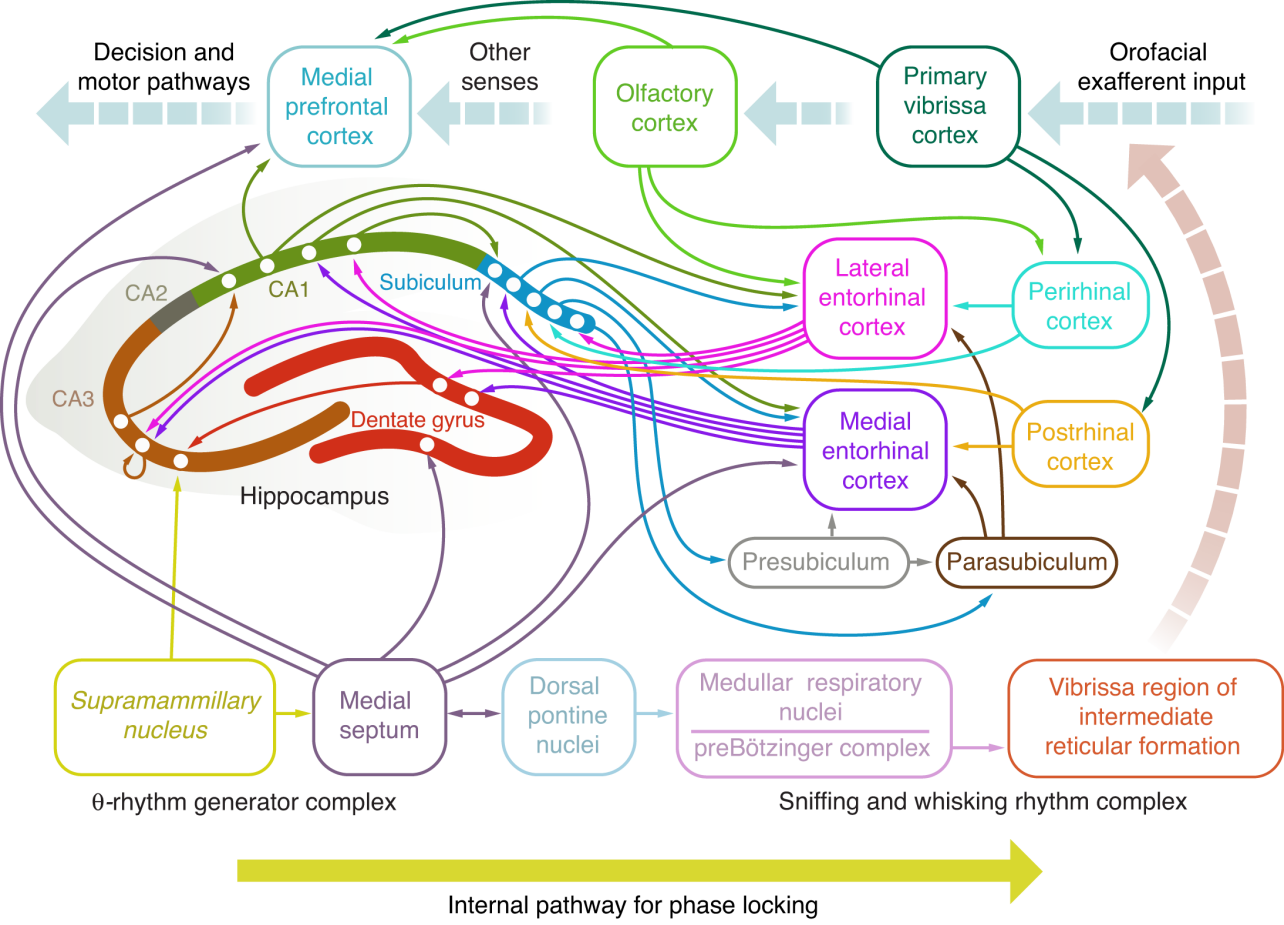
Schematic view of the circuitry of the temporal lobe and its connections to other brain areas of relevance. Figure abstracted from published results [7–15]. Composite illustration by Julia Kuhl.

The specific nature of signal transformation and neuronal computations within the hippocampus is largely an open issue that defines the agenda of a great many laboratories. Equally vexing is the nature of signal transformation as the output leaves the hippocampus and propagates back to regions in the neocortex (Fig 1)—including the medial prefrontal cortex, a site of sensory integration and decision-making—in order to influence perception and motor action. The current experimental data suggest that only some signals within the sensory stream propagate into and out of the hippocampus. What regulates communication with the hippocampus or, more generally, with structures within the temporal lobe? The results from studies in rats and mice suggest that the most parsimonious hypothesis, at least for rodents, involves the rhythmic nature of neuronal activity at the so-called θ-rhythm [16], a 5–10 Hz oscillation (reviewed in [17]). The origin of the rhythm is not readily localized to a single locus [10], but certainly involves input from the medial septum [17] (a member of the forebrain cholinergic system) as well as from the supramammillary nucleus [10,18] (a member of the hypothalamus). The medial septum projects broadly to targets in the hippocampus and entorhinal cortex (Fig 1) [10]. Many motor actions, such as the orofacial actions of sniffing, whisking, and licking, occur within the frequency range of the θ-rhythm [19,20]. Thus, sensory input that is modulated by rhythmic self-motion can, in principle, phase-lock with hippocampal activity at the θ-rhythm to ensure the coherent trafficking of information between the relevant neocortical regions and temporal lobe structures [21–23].

We now shift to the nature of orofacial sensory inputs, specifically whisking and sniffing, which are believed to dominate the world view of rodents [19]. Recent work identified a premotor nucleus in the ventral medulla, named the vibrissa region of the intermediate reticular zone, whose oscillatory output is necessary and sufficient to drive rhythmic whisking [24]. While whisking can occur independently of breathing, sniffing and whisking are synchronized in the curious and aroused animal [24,25], as the preBötzinger complex in the medulla [26]—the oscillator for inspiration—paces whisking at nominally 5–10 Hz through collateral projections [27]. Thus, for the purposes of reviewing evidence for the locking of orofacial sensory inputs to the hippocampal θ-rhythm, we confine our analysis to aroused animals that function with effectively a single sniff/whisk oscillator [28].

What is the evidence for the locking of somatosensory signaling by the vibrissae to the hippocampal θ-rhythm? The first suggestion of phase locking between whisking and the θ-rhythm was based on a small sample size [29,30], which allowed for the possibility of spurious correlations. Phase locking was subsequently reexamined, using a relatively large dataset of 2 s whisking epochs, across many animals, as animals whisked in air [31]. The authors concluded that while whisking and the θ-rhythm share the same spectral band, their phases drift incoherently. Yet the possibility remained that phase locking could occur during special intervals, such as when a rat learns to discriminate an object with its vibrissae or when it performs a memory-based task. This set the stage for a further reexamination of this issue across different epochs in a rewarded task. Work from Diamond's laboratory that is published in the current issue of *PLOS Biology* addresses just this point in a well-crafted experiment that involves rats trained to perform a discrimination task.

Grion, Akrami, Zuo, Stella, and Diamond [32] trained rats to discriminate between two different textures with their vibrissae. The animals were rewarded if they turned to a water port on the side that was paired with a particular texture. Concurrent with this task, the investigators also recorded the local field potential in the hippocampus (from which they extracted the θ-rhythm), the position of the vibrissae (from which they extracted the evolution of phase in the whisk cycle), and the spiking of units in the vibrissa primary sensory cortex. Their first new finding is a substantial increase in the amplitude of the hippocampal field potential at the θ-rhythm frequency—approximately 10 Hz for the data of Fig 2A—during the two, approximately 0.5 s epochs when the animal approaches the textures and whisks against it. There is significant phase locking between whisking and the hippocampal θ-rhythm during both of these epochs (Fig 2B), as compared to a null hypothesis of whisking while the animal whisked in air outside the discrimination zone. Unfortunately, the coherence between whisking and the hippocampal θ-rhythm could not be ascertained during the decision, i.e., turn and reward epochs. Nonetheless, these data show that the coherence between whisking and the hippocampal θ-rhythm is closely aligned to epochs of active information gathering.

**Fig 2:**
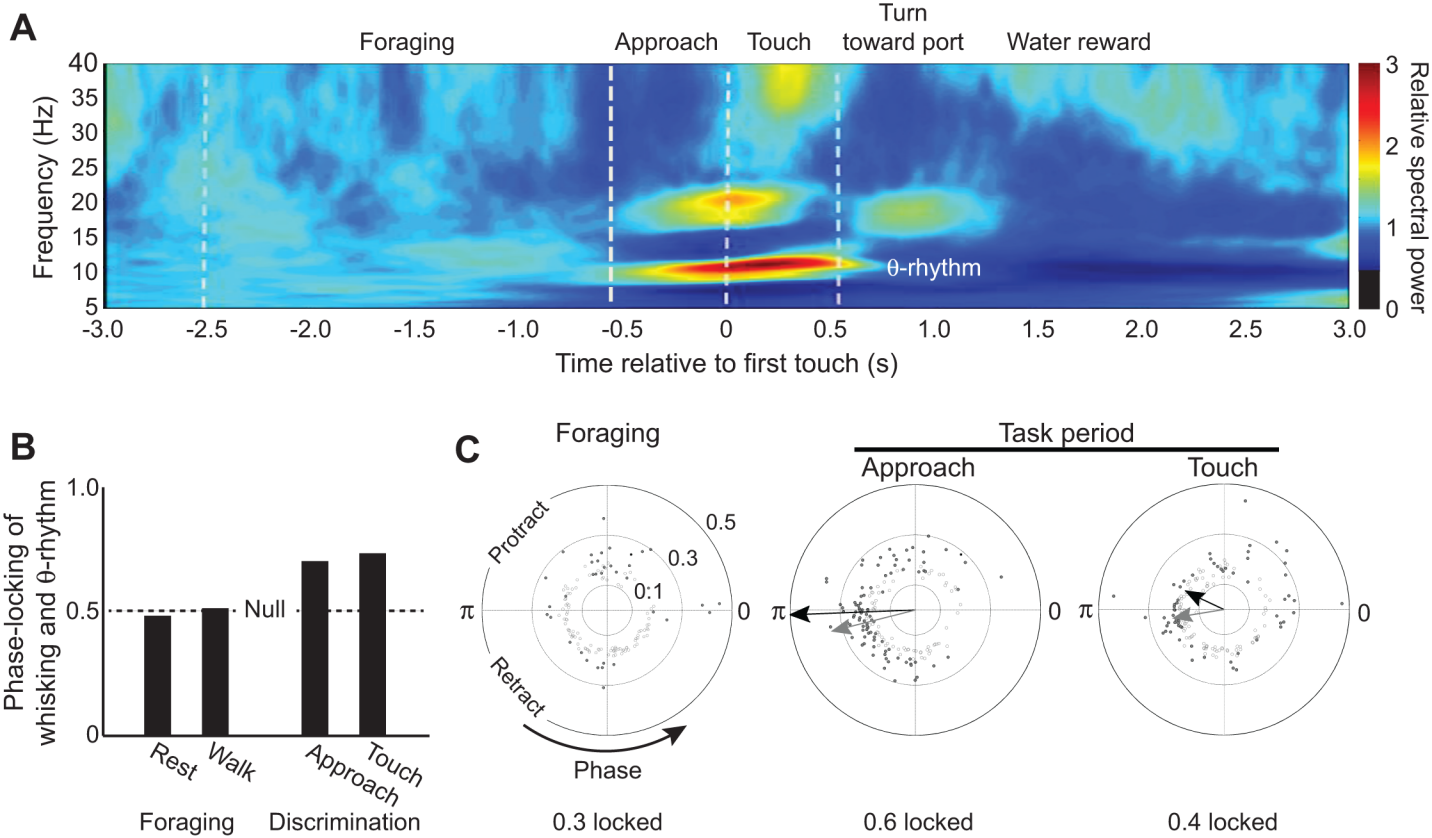
Summary of findings on the θ-rhythm in a rat during a texture discrimination task, derived from reference [32]. **(A)** Spectrogram showing the change in spectral power of the local field potential in the hippocampal area CA1 before, during, and after a whisking-based discrimination task. **(B)** Summary index of the increase in coherence between the band-limited hippocampal θ-rhythm and whisking signals during approach of the rat to the stimulus and subsequent touch. The index reports $\sqrt{{\langle \text{sin}(\phi_{H}-\phi_{W})\rangle }^{2}+{\langle \text{cos}(\phi_{H}-\phi_{W})\rangle }^{2}}$, where ɸ_H_ and ɸ_W_ are the instantaneous phase of the hippocampal and whisking signals, respectively, and averaging is over all trials and animals. **(C)** Summary indices of the increase in coherence between the band-limited hippocampal θ-rhythm and the spiking signal in the vibrissa primary sensory cortex (“barrel cortex”). The magnitude of the index for each neuron is plotted versus phase in the θ-rhythm. The arrows show the concentration of units around the mean phase—black arrows for the vector average across only neurons with significant phase locking (solid circles) and gray arrows for the vector average across all neurons (open and closed circles). The concurrent positions of the vibrissae are indicated. The vector average is statistically significant only for the approach (p < 0.0001) and touch (p = 0.04) epochs.

The second finding by Grion, Akrami, Zuo, Stella, and Diamond [32] addresses the relationship between spiking activity in the vibrissa primary sensory cortex and the hippocampal θ-rhythm. The authors find that spiking is essentially independent of the θ-rhythm outside of the task (foraging in Fig 2C), similar to the result for whisking and the θ-rhythm (Fig 2B). They observe strong coherence between spiking and the θ-rhythm during the 0.5 s epoch when the animal approaches the textures (approach in Fig 2C), yet reduced (but still significant) coherence during the touch epoch (touch in Fig 2C). The latter result is somewhat surprising, given past work from a number of laboratories that observe spiking in the primary sensory cortex and whisking to be weakly yet significantly phase-locked during exploratory whisking [33–37]. Perhaps overtraining leads to only a modest need for the transfer of sensory information to the hippocampus. Nonetheless, these data establish that phase locking of hippocampal and sensory cortical activity is essentially confined to the epoch of sensory gathering.

Given the recent finding of a one-to-one locking of whisking and sniffing [24], we expect to find direct evidence for the phase locking of sniffing and the θ-rhythm. Early work indeed reported such phase locking [38] but, as in the case of whisking [29], this may have been a consequence of too small a sample and, thus, inadequate statistical power. However, Macrides, Eichenbaum, and Forbes [39] reexamined the relationship between sniffing and the hippocampal θ-rhythm before, during, and after animals sampled an odorant in a forced-choice task. They found evidence that the two rhythms phase-lock within approximately one second of the sampling epoch. We interpret this locking to be similar to that seen in the study by Diamond and colleagues (Fig 2B) [32]. All told, the combined data for sniffing and whisking by the aroused rodent, as accumulated across multiple laboratories, suggest that two oscillatory circuits—the supramammillary nucleus and medial septum complex that drives the hippocampal θ-rhythm and the preBötzinger complex that drives inspiration and paces the whisking oscillator during sniffing (Fig 1)—can phase-lock during epochs of gathering sensory information and likely sustain working memory.

What anatomical pathway can lead to phase locking of these two oscillators? The electrophysiological study of Tsanov, Chah, Reilly, and O’Mara [9] supports a pathway from the medial septum, which is driven by the supramammillary nucleus, to dorsal pontine nuclei in the brainstem. The pontine nucleus projects to respiratory nuclei and, ultimately, the preBötzinger oscillator (Fig 1). This unidirectional pathway can, in principle, entrain breathing and whisking. Phase locking is not expected to occur during periods of basal breathing, when the breathing rate and θ-rhythm occur at highly incommensurate frequencies. However, it remains unclear why phase locking occurs only during a selected epoch of a discrimination task, whereas breathing and the θ-rhythm occupy the same frequency band during the epochs of approach, as well as touch-based target selection (Fig 2A). While a reafferent pathway provides the rat with information on self-motion of the vibrissae (Fig 1), it is currently unknown whether that information provides feedback for phase locking.

A seeming requirement for effective communication between neocortical and hippocampal processing is that phase locking must be achieved at all possible phases of the θ-rhythm. Can multiple phase differences between sensory signals and the hippocampal θ-rhythm be accommodated? Two studies report that the θ-rhythm undergoes a systematic phase-shift along the dorsal–ventral axis of the hippocampus [40,41], although the full extent of this shift is only π radians [41]. In addition, past work shows that vibrissa input during whisking is represented among all phases of the sniff/whisk cycle, at levels from primary sensory neurons [42,43] through thalamus [44,45] and neocortex [33–37], with a bias toward retraction from the protracted position. A similar spread in phase occurs for olfactory input, as observed at the levels of the olfactory bulb [46] and cortex [47]. Thus, in principle, the hippocampus can receive, transform, and output sensory signals that arise over all possible phases in the sniff/whisk cycle. In this regard, two signals that are exactly out-of-phase by π radians can phase-lock as readily as signals that are in-phase.

What are the constraints for phase locking to occur within the observed texture identification epochs? For a linear system, the time to lock between an external input and hippocampal theta depends on the observed spread in the spectrum of the θ-rhythm. This is estimated as Δf ~3 Hz (half-width at half-maximum amplitude), implying a locking time on the order of 1/Δf ~0.3 s. This is consistent with the approximate one second of enhanced θ-rhythm activity observed in the study by Diamond and colleagues (Fig 2A) [32] and in prior work [39,48] during a forced-choice task with rodents.

Does the θ-rhythm also play a role in the gating of output from the hippocampus to areas of the neocortex? Siapas, Lubenov, and Wilson [48] provided evidence that hippocampal θ-rhythm phase-locks to electrical activity in the medial prefrontal cortex, a site of sensory integration as well as decision-making. Subsequent work [49–51] showed that the hippocampus drives the prefrontal cortex, consistent with the known unidirectional connectivity between Cornu Ammonis area 1 (CA1) of the hippocampus and the prefrontal cortex [11] (Fig 1). Further, phase locking of hippocampal and prefrontal cortical activity is largely confined to the epoch of decision-making, as opposed to the epoch of sensory gathering. Thus, over the course of approximately one second, sensory information flows into and then out of the hippocampus, gated by phase coherence between rhythmic neocortical and hippocampal neuronal activity.

It is of interest that the medial prefrontal cortex receives input signals from sensory areas in the neocortex [52] as well as a transformed version of these input signals via the hippocampus (Fig 1). Yet it remains to be determined if this constitutes a viable hub for the comparison of the original and transformed signals. In particular, projections to the medial prefrontal cortex arise from the ventral hippocampus [2], while studies on the phase locking of hippocampal θ-rhythm to prefrontal neocortical activity were conducted in dorsal hippocampus, where the strength of the θ-rhythm is strong compared to the ventral end [53]. Therefore, similar recordings need to be performed in the ventral hippocampus. An intriguing possibility is that the continuous phase-shift of the θ-rhythm along the dorsal to the ventral axis of the hippocampus [40,41] provides a means to encode the arrival of novel inputs from multiple sensory modalities relative to a common clock.

A final issue concerns the locking between sensory signals and hippocampal neuronal activity in species that do not exhibit a continuous θ-rhythm, with particular reference to bats [54–56] and primates [57–60]. One possibility is that only the up and down swings of neuronal activity about a mean are important, as opposed to the rhythm per se. In fact, for animals in which orofacial input plays a relatively minor role compared to rodents, such a scheme of clocked yet arrhythmic input may be a necessity. In this case, the window of processing is set by a stochastic interval between transitions, as opposed to the periodicity of the θ-rhythm. This may imply that up/down swings of neuronal activity may drive hippocampal–neocortical communications in all species, with communication mediated via phase-locked oscillators in rodents and via synchronous fluctuations in bats and primates. The validity of this scheme and its potential consequence on neuronal computation remains an open issue and a focus of ongoing research.

## Abbreviations

CA: Cornu Ammonis
Hz: Hertz
s: seconds
